# The Role of SBDP Protein as a Potential Biomarker for Early-Onset Subarachnoid Hemorrhagic

**DOI:** 10.3390/medicina61030454

**Published:** 2025-03-05

**Authors:** Ita M. Sari, Selfy Oswari, Paulus A. Ong, Achmad Adam, Nur Atik

**Affiliations:** 1 Doctoral Program in Medical Sciences, Faculty of Medicine, Universitas Padjadjaran, Bandung 40161, Indonesia; ita.muharram.sari@rspon.co.id (I.M.S.); selfy.oswari@unpad.ac.id (S.O.); 2 National Brain Center Hospital Prof. Dr. dr. Mahar Mardjono, Jakarta 13630, Indonesia; 3 Department of Neurology, Faculty of Medicine, Universitas Padjadjaran, Bandung 40161, Indonesia; anam_ong@yahoo.com; 4 Department of Neurosurgery, Faculty of Medicine, Universitas Padjadjaran, Bandung 40161, Indonesia; adamneurochi@gmail.com; 5 Department of Biomedical Sciences, Faculty of Medicine, Universitas Padjadjaran, Bandung 40161, Indonesia

**Keywords:** SAH, clinical outcome, SBDP, spectrin, vasospasm

## Abstract

*Background and Objectives:* Cerebral vasospasm is the most common complication of subarachnoid hemorrhage (SAH) that is related to high mortality and morbidity. Early biomarkers predicting those conditions are still limited. This study aims to analyze spectrin degradation products (SBDPs) as potential biomarkers for SAH patients, which can be used to monitor clinical outcomes. *Materials and Methods:* We conducted a prospective observational study in acute SAH within 72 h of onset. All patients underwent placement of continuous cerebrospinal drainage, and liquor was taken four times and analyzed using ELISA to measure SBDP150, SBDP145, and SBDP120 levels and analyzed using Friedman test and post hoc Wilcoxon analysis. The relationship between SBDP levels and vasospasm, as well as functional outcomes (using the Glasgow Outcome Scale–Extended, GOSE), was assessed. *Results:* We enrolled thirty-five patients: thirty patients with lumbar drainage (LD) and five with extra ventricular drainage (EVD). Friedman’s analysis showed significant changes over time for SBDP120 (*p* = 0.0001) and SBDP145 (*p* = 0.0001), but not for SBDP150 (*p* = 0.218). Levels of SBDP120 on day 3 (*p* = 0.001), SBDP120 on day 5 (*p* = 0.022), and SBDP145 on day 3 (*p* = 0.005) in EVD group were higher than in the LD group. SBDP145 on day 5 was significantly higher in patients with vasospasm (*p* = 0.041 in all patients, *p* = 0.028 in LD patients), indicating its potential as an early biomarker for vasospasm. SBDP145 on day 7 (*p* = 0.014) is the strongest predictor of unfavorable GOSE at 90 days in all patients. In LD patients, SBDP145 on day 7 (*p* = 0.002), SBDP120 on day 7 (*p* = 0.009), and SBDP120 on day 10 (*p* = 0.043) were significantly associated with poor GOSE at 90 days. *Conclusions:* A higher level of SBDP145 on day 5 can predict vasospasm risk, while an elevated level of SBDP145 and SBDP120 on day 7 is a potential predictor of poor functional outcomes. SBDPs may serve as valuable biomarkers for SAH management.

## 1. Introduction

Subarachnoid hemorrhage (SAH) is a kind of stroke that is related to high mortality and morbidity. A ruptured aneurysm is the most common cause of SAH, with a high risk of re-bleeding. Early endovascular coiling or surgical clipping has already been performed to prevent the high-risk incidence of re-bleeding. Cerebral vasospasm or delayed cerebral ischemia remains the most significant cause of complications and, thereby, poor neurological outcomes in patients [1,2,3].

The previous literature has proposed various pathophysiological mechanisms explaining cerebral vasospasms, but the definite mechanism that induces cerebral vasospasm is not yet sufficiently precise. Thus, the optimal management of cerebral vasospasm is not fully established [3,4]. Identifying biological markers in CSF may provide early warning of ischemia from cerebral vasospasm and could offer more therapy for patients [5].

Continued bloody cerebrospinal fluid drainage (CCFD) strategies have been the subject of recent research, considering their impact on the clearance of intraventricular and subarachnoid blood with consequent effects on cerebral vasospasm and clinical outcomes. Among CCFD procedures, external ventricular drainage (EVD) and lumbar drainage (LD) are currently the most widely used techniques [6,7]. Previous studies suggest that LD placement is promising for preventing cerebral vasospasm and gives better clinical outcomes [8,9].

αII-spectrin is an essential membrane cytoskeleton component that provides membrane structure and integrity [10]. During acute brain injury, including SAH, protease degrades αII-spectrin and becomes spectrin breakdown products (SBDPs). Specifically, calpain and caspase-3 will produce the initial fragment SPBD 150 kDa, then SBDP 145 kDa by calpain, and SBDP 120 kDa by caspase-3. In previous studies, significant increases in SBDP levels were significantly correlated with cerebral vasospasm, delayed cerebral ischemia, and poor outcomes [11,12,13]. The increase in SBDP levels is mainly thought to be due to necrosis [11]. However, several studies recently show that apoptosis plays an important role in the pathogenesis of secondary brain injury after SAH.

Studies regarding the utility of SBDPs as biomarkers in SAH are still limited. Also, no study has assessed the relationship between SBDP levels in SAH patients with LD placement. This study aimed to assess the diagnosis and prognosis utility of SBDPs as a biomarker by examining levels of SBDP120, SBDP145, and SBDP150 in SAH patients with EVD and LD. It also assessed the relationship between SBDP levels and vasospasm, GOSE at discharge, and GOSE at 90 days.

## 2. Materials and Methods

### 2.1. Design and Setting

We performed a prospective observational study at the National Brain Center Hospital Prof. Dr. dr. Mahar Mardjono, Jakarta, from August 2021 to December 2023. The National Brain Center Ethical Research Committee approved the study (LB.02.01/KEP/048/2021).

### 2.2. Recruitment

The inclusion criteria were acute SAH patients admitted within 72 h of onset, age 40 up to 70 years old, with a World Federation of Neurological Surgery Scale (WFNS) score grade 1–4. Exclusion criteria were patients with diabetes, traumatic subarachnoid bleeding, a history of meningitis or encephalitis, Parkinson’s disease, Alzheimer’s, and contraindications of EVD or LD placement. Based on clinical adjustment by the neurologist onsite, the patient will consult for EVD or LD placement. We used diagnosed criteria as defined in the Tirilazad Trials to define the criteria of cerebral vasospasm [6]. For diagnostic vasospasm, we used serial TCD examinations or a cerebral DSA procedure. We used the Glasgow Outcome Score Extended (GOSE) score, which ranges from 1 to 8, to assess patients’ clinical outcomes at discharge and 90 days after stroke [14].

### 2.3. SBDP Analysis

Cerebrospinal fluid samples were collected 4 times: the first samples between 48 and 72 h onset (day 3), the second on day 5, the third on day 7, and the fourth on day 10. CSF samples were centrifuged and frozen at −80 °C. Samples were coded using a bar code catalog system to assure patient confidentiality. SBDPs were measured using ELISA. We used the following measurement kits: Human SBDP 145 (αII SBDP) Elisa Kit (Nordic Biosite EKX-OUYZAN, Täby, Sweden), Human SBDP 120 (αII SBDP) Elisa Kit (Nordic Biosite EKX-4PF4P4), Human SBDP 150 (αII SBDP) Elisa Kit (Nordic Biosite EKX-ROXRYQ).

### 2.4. Data Analysis

Using a Mann–Whitney test, we presented baseline characteristics, including WFNS score, Fisher grade, GOSE at discharge, and GOSE at 90 days. For age, we used an independent t-test. For categorical data, including sex, vasospasm, and GOSE categorical, we used an exact Fisher test. We used the Friedman test to compare each level of SBDPs, then continued with the Wilcoxon test for post hoc analysis. Then, we used a Mann–Whitney test to analyze the comparison between levels of SBDP with vasospasm, GOSE at discharge, GOSE at 90 days, and SBDP level between EVD and LD. We used STATA (version 17.0), Stata Corp., College Station, TX, USA, for statistical analysis.

## 3. Results

This section may be divided by subheadings. It should provide a concise and precise description of the experimental results, their interpretation, as well as the experimental conclusions that can be drawn.

### 3.1. Baseline Characteristics

Thirty-five patients were included in this study, including thirty patients with LD and five with EVD. The baseline characteristics of patients are listed in Table 1.

### 3.2. Average SBDPs Level

Patients were enrolled within 72 h of onset. Cerebrospinal fluid samples were collected on days 3, 5, 7, and 10. Initially, we analyzed the mean SBDP level in all patients at each sampling time. Table 2 calculates the comparison level of SBDP120, SBDP145, and SBDP150 in all patients on day 3, day 5, day 7, and day 10.

The results of Friedman’s analysis regarding comparative levels of SBDP, which area shown in Table 2, indicate statistical significance for SBDP120 and SBDP145 across different time points (*p* = 0.0001). As a result, we performed with a post hoc analysis using the Wilcoxon test, to determine the specific time points that were significantly different from each other. The findings of the post hoc analysis are presented in Table 3.

Based on the post hoc results in Table 3, we can observe there are significant differences in the level of SBDP120 in the following: day 3 vs. day 5, day 3 vs. day 7, day 3 vs. day 10, day 5 vs. day 7, and day 5 vs. day 10 in all patients. The level of SBDP120 indicates stability after day 7. We also identified significant differences in the level of SBDP145 in the following: day 3 vs. day 5, day 3 vs. day 7, and day 3 vs. day 10 in all patients. However, we were unable to find differences in each level of SBDP150, confirming that SBDP150 remained stable. Then, in Table 4, we compared each SBDP120, SBDP145, and SBDP150 levels in patients with LD.

Similarly, in Table 2, the results of Friedman’s analysis for the comparative levels of SBDP in Table 4 also showed statistical significance for SBDP120 and SBDP145 (*p* < 0.05). The *p*-value for SBDP145 is slightly higher in LD group (*p* = 0.003) than in the total sample (*p* = 0.0001), suggesting that changes in SBDP145 are less pronounced in LD patients. We conducted post hoc analysis using the Wilcoxon test, with the results displayed in Table 5.

According to the post hoc results presented in Table 5, we can observe significant differences in the level of SBDP120 when comparing day 3 to day 5, day 3 to day 7, day 3 to day 10, and day 5 to day 7 in LD patients. Additionally, we also identified significant differences in the level of SBDP145 on day 3 vs. day 5, day 3 vs. day 7, and day 3 vs. day 10 in LD patients. SBDP145 levels in the LD group declined more gradually, leading to weaker statistical significance in post hoc tests. However, we were also unable to find differences in the levels of SBDP150 among LD patients.

### 3.3. SBDPs Level Between EVD and LD

We compared each level of SBDP120, SBDP145, and SBDP150 between EVD and LD groups, as shown in Table 6 below.

The results in Table 6 above show that the level of SBDP120 on day 3 (*p* = 0.001), SBDP120 on day 5 (*p* = 0.022), and SBDP145 on day 3 (*p* = 0.005) in the EVD group were higher than in the LD group. There are no differences in the level of SBDP150 between two groups.

### 3.4. SBDPs Level and Cerebral Vasospasm 

In Table 7, we outline a comparison of levels of SBDP based on vasospasm in all patients and in LD group patients.

The results in Table 7 above show that patients with vasospasm had significantly higher levels of SBDP 145 on day 5 than patients without vasospasm, both in the total sample (*p* = 0.041) and the lumbar drain group (*p* = 0.028).

### 3.5. SBDPs Level and Clinical Outcome

We used the GOSE (Glasgow Outcome Scale-Extended) score to assess clinical outcomes at discharge and 90 days. The GOSE scores were divided into favorable (category 5–8) and unfavorable (category 1–4). We compared the SBDP levels between GOSE at discharge and at 90 days in all patients, and these are listed in Table 8.

Based on the results shown in Table 8 above, there is no correlation between each level of SBDP and GOSE at discharge in all patients. Based on GOSE at 90 days, we found that a high level of SBDP145 on day 7 (*p* = 0.014) correlates with unfavorable outcomes. Then, we compared each level of SBDP with GOSE at discharge and at 90 days in LD groups, as shown below in Table 9.

The results from Table 9 show that LD patients with an unfavorable GOSE at discharge have a high level of SBDP120 on day 7 (*p* = 0.015) and SBDP145 on day 7 (*p* = 0.009). Based on GOSE at 90 days, LD patients with an unfavorable GOSE have higher levels of SBDP120 on day 7 (*p* = 0.009), SBDP120 on day 10 (*p* = 0.043), and SBDP145 on day 7 (*p* = 0.002) than favorable GOSE patients.

## 4. Discussion

Although the incidence of subarachnoid hemorrhage is less than another stroke, the consequences are more devastating. High prehospital mortality and some complications like cerebral vasospasm, seizure, and hydrocephalus affect the clinical outcomes of patients. Studies about biomarkers in SAH, especially vasospasm-related ones, are still minimal [5,13]. Based on the results of this study, we have matched participants between the EVD and LD groups, because differences in participant characteristics influence the results.

αII-spectrin is a major axonal cytoskeletal protein that will degrade into spectrin breakdown products (SBDPs) when axons get injured. SBDPs have unique abilities from other biomarkers to identify two central proteolytic cascades [15,16]. Calpain that relates to necrotic cells will activate and cleave aII-spectrin (280 kDa) into 150 kDa (SBDP150) and 145 kDa (SBDP145) fragments [15,17]. Then, caspase-3, which is related to apoptosis cells, will cleave spectrin to become a 120 kDa (SBDP120) fragment [15]. Other studies also mention that SBDP150 will be formed due to caspase-3 activation [18]. These SBDPs can be isolated from cerebrospinal liquor, whose injury severity is highly correlated with the quantitative and temporal expression of SBDP. It is also very important that spectrin breakdown faithfully reflects axonal damage.

### 4.1. Average SBDPs Level

Our study found that levels of SBDP120 and SBDP145 significantly changed over time, while SBDP150 did not. Calpain is activated in various necrotic and apoptotic conditions, while caspase-3 is only activated in neuronal apoptosis [15]. Necrotic and apoptotic pathways often co-occur and depend on the strength of the initial injury [3,4]. However, unlike other strokes, necrosis is not a significant factor in SAH [3,4]. We thought that SBDP120 levels that increased significantly on day 3 were due to apoptosis in endothelial cells that had appeared since the onset of SAH. Unlike other acute brain injuries, patients with acute SAH will suffer early brain injury (EBI) that occurs before 72 h of onset [3,4].

Previous studies have proved that SBDP150 might be associated with calpain and caspase activity [14]. However, cleavage caspase for SBDP150 is unusually efficient, so it may cause a low level of SBDP150 in our study and not interfere with clinical outcomes [18]. In a previous study in TBI, SBDP150 will appear first after injury and a significant eightfold increase in 3 h post-injury [17]. Further processing fragment was then followed by an increased level of SBDP145 with maximal accumulation (30-fold increase) at 48 h post-injury [17]. Our post hoc Wilcoxon tests revealed that SBDP120 and SBDP145 levels were significantly lower after day 3, indicating that neuronal injury markers decrease as recovery progresses. One study showed that increased levels of SBDPs return to near-basal levels of spectrin (240 kDa) 14 days post-injury [17]. Maximal accumulations of SBDP145 were evident 5 to 14 days post-injury [17].

### 4.2. SBDPs Level Between EVD and LD

Our study shows that the levels of SBDP120 on day 3 (*p* = 0.001), SBDP120 on day 5 (*p* = 0.022), and SBDP145 on day 3 (*p* = 0.005) in the EVD group were significantly higher than those in the LD group. This elevated level suggests that severe neuronal injury, mediated by calpain or caspase-3 in early-phase SAH, was more pronounced in the EVD group than in the LD group. In EVD, a drainage catheter is inserted into cerebral ventricles, allowing most SBDPs to flow through these ventricles. Lumbar drainage is suggested to promote clear circulation, including red cell mass, from the intra thecal space rather than through EVD. A previous study also suggests that delayed washout is a mechanism that keeps SBDP145’s level high in EVD, in addition to continued protein degradation or prolonged half-life [11]. The differences in biomarker levels between the two groups disappear by day 7 and day 10, suggesting that the injury response stabilizes over time.

### 4.3. SBDPs Level and Vasospasm Cerebral

Our study of patients shows that patients with vasospasm had significantly higher levels of SBDP 145 on day 5 than patients without vasospasm, both in the total sample (*p* = 0.041) and lumbar drain group (*p* = 0.028). This result follows a previous study showing that all three SBDP levels significantly increased in patients with vasospasm, especially SBDP150 and SBDP145, which are predominant in necrosis activity [11].

EBI that occurs within 72 h after SAH onset could develop into delayed cerebral ischemia or cerebral vasospasm [3,4]. Oxidative stress produces free radicals, causes cellular damage and protein breakdown, and leads to pathological changes between endothelial cells, widening inter-endothelial spaces, cellular apoptosis, necrosis, and increased BBB permeability [3,4]. These morphological changes will improve with time following SAH and reach a peak on days 5 and 7 after onset, which correlates with the development of cerebral vasospasm [3]. It is well known that calpain is also activated in some apoptosis systems [15]. Both necrosis and apoptosis processes continue in acute SAH, mainly if vasospasm occurs [15]. That way, SBDP145 levels will remain high in vasospasm patients. Our findings suggest that SBDP145 on day 5 is a strong predictor of vasospasm, regardless of drainage type. Patients with increased level of SBDP145 on day 5 would require close monitoring and possible early intervention.

### 4.4. SBDP Level and Clinical Outcome

When we compare clinical outcomes in all patients, based on GOSE at 90 days, we found that high-level SBDP145 on day 7 (*p* = 0.014) correlated with unfavorable outcomes. In LD groups, patients with unfavorable GOSE at discharge have a high level of SBDP120 on day 7 (*p* = 0.015) and SBDP145 on day 7 (*p* = 0.009). Based on GOSE at 90 days, LD patients with unfavorable GOSE have higher levels of SBDP120 on day 7 (*p* = 0.009), SBDP120 on day 10 (*p* = 0.043), and SBDP145 on day 7 (*p* = 0.002) than favorable GOSE patients.

Even though caspase-3 does not need Ca^2+^ for initiated activity, higher concentrations of Ca^2+^, like in EBI, can lead to caspase-mediated apoptosis [15]. The apoptosis process that happens earlier in day 3 will correlate with poor clinical outcomes. OxyHb, oxidative stress and excess iron also contribute to endothelial cell apoptosis [4]. The apoptosis cascade will disrupt the blood–brain barrier, induce brain edema, decrease the production of endothelial-dependent relaxing factors, and aggravate vasospasm [4]. Due to global ischemic ischemia, secondary to raised ICP and decreased CBF soon after onset, apoptosis shows as widespread in the brain after SAH [3,4]. Several apoptotic pathways are believed to play a role in SAH, including active caspase-dependent and caspase-independent pathways, and SBDP120 increases earlier after acute SAH [3,4]. In patients with unfavorable outcomes, the level of SBDP120 on day 10 is still high due to the continuous apoptotic process. Our findings suggest that SBDP145 levels on day 7 might be useful for predicting long-term functional outcomes in SAH patients. SBDP120 on days 7 and 10 is significantly associated with worse outcomes in LD patients. These findings suggest that monitoring SBDP120 and SBDP145, particularly on day 7, could provide valuable prognostic information for SAH patients, guiding treatment strategies and rehabilitation plans.

### 4.5. Strengths and Limitations

Studies related to SBDP biomarkers in SAH are minimal. This is the first study in which liquor was collected from a lumbar drain. Based on the current trial, lumbar drainage is a promising treatment for lessening the burden of secondary infarction and decreasing the rate of unfavorable outcomes [9,19]. Our study has some limitations, including a small sample size and uneven distribution between LD and EVD. The small number of EVD patients may limit the generalization of our findings. This study was conducted in a single center at a national referral hospital, where EVD procedures are infrequent, which further limits the external validity and the applicability of the results to other populations. For follow-up of clinical outcomes, we only assessed for up to 90 day, and this may not fully capture long-term neurological recovery in SAH patients. Our study did not analyze some confounding factors, including initial SAH severity and patient comorbidities, which could influence SBDP levels and clinical outcomes. In addition, this study cannot determine the baseline standard value of each SBDP in vasospasm or poor prognosis.

### 4.6. Further Directions

The use of SBDP as a biomarker in SAH patients is promising. Further research is needed, including a larger, more diverse patient population, to validate the predictive role of SBDP145 for vasospasm and SBDP120 for clinical outcomes. Research for validation of SBDP cut-off values is also important, as they can guide early therapeutic interventions, such as those accompanied by anti-calpain and anti-caspase treatment from the beginning of onset.

## 5. Conclusions

SBDP120 and SBDP145 are relevant biomarkers for assessing neuronal damage in SAH patients. SBDP120 and SBDP145 in the EVD group have higher levels than those in the LD group. Vasospasm is linked to higher SBDP145 levels on day 5, and then elevated SBDP120 and SBDP145 levels on day 7 are associated with poorer functional recovery, making them potential prognostic markers. SBDP150 does not significantly change over time, nor correlate with clinical outcomes, indicating limited relevance in SAH monitoring. SBDP120 and SBDP145 is promising for biomarkers in SAH patients, particularly in relation to vasospasm and long-term functional outcome.

## Figures and Tables

**Table 1:** Baseline characteristics of patients between EVD and LD group.

Variables	CSF Drainage Type (*n* = 35)		*p*-Value
	LD (*n* = 30)	EVD (*n* = 5)	
Age, mean ± SD	56.20 ± 8.79	67.40 ± 1.14	0.008 ^a^*
Female, number; %	13 (43.3%)	4 (80.0%)	0.177 ^b^
WFNS score, median (IQR)	3 (1–4)	4 (2–4)	0.321 ^c^
m-Fisher grade, median (IQR)	3 (1–4)	3 (3–4)	0.095 ^c^
Vasospasm, number; %	9 (30%)	1 (20.0%)	1.000 ^b^
GOSE at discharge, median (IQR)	3.5 (1–8)	4 (1–7)	0.664 ^c^
Favorable GOSE at discharge, number; %	14 (46.7%)	2 (40.0%)	1.000 ^b^
GOSE at 90 days, median (IQR)	3.5 (1–8)	7 (1–8)	0.664 ^c^
Favorable GOSE at 90 days, number; %	14 (46.7)	3 (60.0%)	0.658 ^b^

^a^ analyzed using independent *t* test; ^b^ analyzed using Fisher test; ^c^ analyzed using Mann–Whitney test; * statistically significant at *p* < 0.05.

**Table 2:** Comparison of SBDPs levels in all patients on day 3, day 5, day 7, and day 10 (*n* = 35).

Variables SBDP	Sampling SBDPs				*p*-Value
	Day 3	Day 5	Day 7	Day 10	
SBDP120	1.93 (0.00–40.00)	0.78 (0.18–38.76)	0.42 (0.00–7.99)	0.36 (0.00–8.84)	0.0001 ^a^*
SBDP145	28.81(0.00–80.00)	6.15 (1.47–80.00)	7.11 (0.00–80.00)	3.68 (0.00–80.00)	0.0001 ^a^*
SBDP145	0.69 (0.00–1.91)	0.73 (0.40–1.87)	0.59 (0.00–3.99)	0.67 (0.00–2.81)	0.218 ^a^

^a^ analyzed using Friedman test; * statistically significant at *p* < 0.05.

**Table 3:** Comparison of SBDPs over time in all patients (*n* = 35).

Variables	*p*-Value ^a^		
	SBDP120 (*n* = 35)	SBDP145 (*n* = 35)	SBDP150 (*n* = 35)
Day 3 vs. day 5	0.003 *	0.002 *	0.499
Day 3 vs. day 7	0.0001 *	0.006 *	0.464
Day 3 vs. day 10	0.001 *	0.002 *	0.911
Day 5 vs. day 7	0.001 *	0.367	0.765
Day 5 vs. day 10	0.009 *	0.077	0.362
Day 7 vs. day 10	0.789	0.177	0.428

^a^ analyzed using Wilcoxon test; * statistically significant at *p* < 0.05.

**Table 4:** Comparison of SBDPs levels in all patients on day 3, day 5, day 7, and day 10 (*n* = 30).

Variables SBDP	Sampling SBDPs				*p*-Value
	Day 3	Day 5	Day 7	Day 10	
SBDP120	0.66 (0.00–21.02)	0.50 (0.18–10.68)	0.38 (0.00–4.02)	0.36 (0.00–8.84)	0.0001 ^a^*
SBDP145	20.51(0.00–80.00)	4.92 (1.47–80.00)	7.12 (0.00–80.00)	3.73 (0.00–80.00)	0.003 ^a^*
SBDP145	0.69 (0.00–1.91)	0.73 (0.40–1.87)	0.59 (0.00–3.99)	0.69 (0.00–2.07)	0.115 ^a^

^a^ analyzed using Friedman test; * statistically significant at *p* < 0.05.

**Table 5:** Comparison of SBDPs over time in all patients (*n* = 30).

Variables	*p*-Value ^a^		
	SBDP120 (*n* = 30)	SBDP145 (*n* = 30)	SBDP150 (*n* = 30)
Day 3 vs. day 5	0.005 *	0.008 *	0.689
Day 3 vs. day 7	0.0001 *	0.045 *	0.241
Day 3 vs. day 10	0.009 *	0.025 *	0.948
Day 5 vs. day 7	0.005 *	0.754	0.921
Day 5 vs. day 10	0.043	0.336	0.368
Day 7 vs. day 10	0.983	0.157	0.361

^a^ analyzed using Wilcoxon test; * statistically significant at *p* < 0.05.

**Table 6:** Comparison each levels of SBDPs between EVD and LD.

Variables	CSF Drainage Type (*n* = 35)		*p*-Value
	LD (*n* = 30)	EVD (*n* = 5)	
SBDP120			
Day 3	0.66 (0.00–21.02)	8.99 (7.64–40.00)	0.001 ^a^*
Day 5	0.50 (0.18–10.68)	2.11 (0.78–38.76)	0.022 ^a^*
Day 7	0.38 (0.00–4.02)	1.15 (0.00–7.99)	0.345 ^a^
Day 10	0.36 (0.00–8.84)	0.83 (0.00–1.34)	1.000 ^a^
SBDP145			
Day 3	20.51 (0.00–80.00)	80.00 (48.15–80.00)	0.005 ^a^*
Day 5	4.92 (1.47–80.00)	62.92 (3.33–80.00)	0.077 ^a^
Day 7	7.12 (0.00–80.00)	5.77 (0.00–80.00)	0.945 ^a^
Day 10	3.73 (0.00–80.00)	3.56 (0.00–36.23)	0.536 ^a^
SBDP150			
Day 3	0.69 (0.00–1.91)	0.82 (0.51–1.39)	1.000 ^a^
Day 5	0.73 (0.40–1.87)	0.65 (0.46–1.25)	1.000 ^a^
Day 7	0.59 (0.00–3.99)	0.75 (0.00–2.36)	0.802 ^a^
Day 10	0.69 (0.00–2.07)	0.47 (0.00–2.81)	0.766 ^a^

^a^ analyzed using Mann–Whitney test; * statistically significant at *p* < 0.05.

**Table 7:** Comparison levels of SBDPs with vasospasm in all patients and LD patients.

Variables	All Patients (*n* = 35)			LD Patients (*n* = 30)		
	Vasospasm (*n* = 10)	No Vasospasm (*n* = 25)	*p*-Value	Vasospasm (*n* = 9)	No Vasospam (*n* = 21)	*p*-Value
SBDP120						
Day 3	1.76 (0.00–16.89)	1.93 (0.21–40.00)	0.843 ^a^	0.57 (0.00–12.43)	0.75 (0.21–21.02)	0.965 ^a^
Day 5	1.43 (0.28–38.76)	0.58 (0.18–20.40)	0.162 ^a^	1.21 (0.28–10.68)	0.36 (0.18–5.98)	0.137 ^a^
Day 7	0.28 (0.00–2.55)	0.42 (0.07–7.99)	0.418 ^a^	0.29 (0.00–2.55)	0.39 (0.17–4.02)	0.965 ^a^
Day 10	0.28 (0.00–1.73)	0.54 (0.00–8.84)	0.070 ^a^	0.29 (0.00–1.73)	0.41 (0.00–8.84)	0.164 ^a^
SBDP145						
Day 3	53.48 (0.00–80.00)	26.35 (1.23–80.00)	0.483 ^a^	52.09 (0.00–80.00)	8.23 (1.23–80.00)	0.326 ^a^
Day 5	20.73 (2.16–80.00)	4.23 (1.47–80.00)	0.041 ^a^*	11.32 (2.16–80.00)	3.98 (1.47–80.00)	0.028 ^a^*
Day 7	9.06 (0.00–73.74)	6.50 (1.85–80.00)	0.760 ^a^	9.32 (0.00–73.74)	6.50 (1.85–80.00)	0.790 ^a^
Day 10	3.65 (0.00–77.49)	3.68 (0.00–80.00)	0.506 ^a^	3.78 (0.00–77.49)	3.68 (0.00–80.00)	0.756 ^a^
SBDP150						
Day 3	0.63 (0.00–1.32)	0.75 (0.53–1.91)	0.059 ^a^	0.65 (0.00–1.32)	0.75 (0.53–1.91)	0.178 ^a^
Day 5	0.84 (0.48–1.49)	0.65 (0.40–1.87)	0.174 ^a^	0.82 (0.48–1.49)	0.71 (0.40–1.87)	0.349 ^a^
Day 7	0.54 (0.00–1.57)	0.61 (0.43–3.99)	0.162 ^a^	0.54 (0.00–1.57)	0.61 (0.43–3.99)	0.422 ^a^
Day 10	0.59 (0.00–1.79)	0.71 (0.00–2.81)	0.358 ^a^	0.67 (0.00–1.79)	0.71 (0.00–2.07)	0.657 ^a^

^a^ analyzed using Mann–Whitney test; * statistically significant at *p* < 0.05.

**Table 8:** Comparison of SBDPs levels with GOSE at discharge and at 90 days in all patients (*n* = 35).

Variables	GOSE at Discharge			GOSE at 90 days		
	Unfavorable GOSE (*n* = 19)	Favorable GOSE (*n* = 16)	*p*-Value	Unfavorable GOSE (*n* = 18)	Favorable GOSE (*n* = 17)	*p*-Value
SBDP120						
Day 3	3.40 (0.00–21.02)	0.46 (0.26–40.00)	0.102 ^a^	3.30 (0.00–21.02)	0.51 (0.26–40.00)	0.245 ^a^
Day 5	0.83 (0.18–38.76)	0.34 (0.23–20.40)	0.193 ^a^	1.24 (0.18–38.76)	0.45 (0.23–20.40)	0.232 ^a^
Day 7	0.73 (0.00–4.02)	0.31 (0.00–7.99)	0.082 ^a^	0.78 (0.00–4.02)	0.26 (0.07–7.99)	0.083 ^a^
Day 10	0.70 (0.00–8.84)	0.29 (0.00–1.73)	0.142 ^a^	0.57 (0.00–8.84)	0.29 (0.00–1.73)	0.096 ^a^
SBDP145						
Day 3	32.76 (0.00–80.00)	26.37 (1.91–80.00)	0.441 ^a^	30.79 (0.00–80.00)	26.38 (1.91–80.00)	0.987 ^a^
Day 5	9.47 (1.65–80.00)	5.68 (1.47–80.00)	0.385 ^a^	7.98 (1.65–80.00)	6.15 (1.47–80.00)	0.369 ^a^
Day 7	14.04 (0.00–80.00)	4.81 (1.85–80.00)	0.056 ^a^	14.27 (0.00–80.00)	4.05 (1.85–80.00)	0.014 ^a^*
Day 10	4.51 (0.00–80.00)	3.26 (0.00–16.77)	0.095 ^a^	12.79 (0.00–80.00)	3.51 (0.00–16.77)	0.062 ^a^
SBDP150						
Day 3	4.51 (0.00–80.00)	3.26 (0.00–16.77)	0.909 ^a^	0.67 (0.00–1.91)	0.75 (0.50–1.57)	0.590 ^a^
Day 5	0.75 (0.00–1.91)	0.69 (0.50–1.57)	0.883 ^a^	0.74 (0.46–1.87)	0.63 (0.40–1.57)	0.405 ^a^
Day 7	0.73 (0.46–1.49)	0.64 (0.40–1.87)	0.806 ^a^	0.60 (0.00–3.99)	0.59 (0.00–2.36)	0.732 ^a^
Day 10	0.84 ± 0.727	1.04 ± 1.012	0.526 ^b^	0.77 (0.00–2.81)	0.61 (0.00–1.91)	0.443 ^a^

^a^ analyzed using Mann–Whitney test; ^b^ analyzed using independent T test; * statistically significant at *p* < 0.05.

**Table 9:** Comparison of SBDPs levels with GOSE at discharge and at 90 days in LD patients (*n* = 30).

Variables	GOSE at Discharge			GOSE at 90 days		
	Unfavorable GOSE (*n* = 16)	Favorable GOSE (*n* = 14)	*p*-Value	Unfavorable GOSE (*n* = 16)	Favorable GOSE (*n* = 14)	*p*-Value
SBDP120						
Day 3	3.01 (0.00–21.02)	0.43 (0.26–3.74)	0.070 ^a^	3.01 (0.00–21.02)	0.46 (0.26–3.74)	0.093 ^a^
Day 5	0.71 (0.18–5.98)	0.32 (0.23–10.68)	0.154 ^a^	0.71 (0.18–5.98)	0.34 (0.23–10.68)	0.193 ^a^
Day 7	0.78 (0.20–4.02)	0.26 (0.00–2.55)	0.015 ^a^*	0.78 (0.20–4.02)	0.26 (0.00–2.55)	0.009 ^a^*
Day 10	0.57 (0.00–8.84)	0.29 (0.00–1.73)	0.142 ^a^	0.57 (0.00–8.84)	0.29 (0.00–1.73)	0.043 ^a^*
SBDP145						
Day 3	21.74 (0.00–80.00)	17.05 (1.91–80.00)	0.637 ^a^	21.74 (0.00–80.00)	19.70 (1.91–80.00)	0.951 ^a^
Day 5	7.05 (1.65–80.00)	4.72 (1.47–80.00)	0.313 ^a^	4.59 (1.65–80.00)	5.68 (1.47–80.00)	0.473 ^a^
Day 7	14.27 (1.63–80.00)	3.80 (0.00–63.28)	0.009 ^a^*	14.27 (1.94–80.00)	2.99 (0.00–63.28)	0.002 ^a^*
Day 10	12.79 (0.00–80.00)	0.00 (0.00–16.77)	0.506 ^a^	12.79 (0.00–80.00)	3.26 (0.00–16.77)	0.064 ^a^
SBDP150						
Day 3	0.78 (0.00–1.91)	0.67 (0.50–1.57)	0.423 ^a^	0.72 (0.00–1.91)	0.69 (0.40–1.57)	0.854 ^a^
Day 5	0.73 (0.46–1.49)	0.72 (0.40–1.87)	0.918 ^a^	0.73 (0.46–1.87)	0.72 (0.40–1.57)	0.951 ^a^
Day 7	0.60 (0.43–3.36)	0.57 (0.00–3.99)	0.498 ^a^	0.60 (0.43–3.99)	0.57 (0.00–1.79)	0.400 ^a^
Day 10	0.91 ± 0.706	0.75 ± 0.510	0.507 ^b^	0.94 ± 0.672	0.71 ± 0.547	0.326 ^b^

^a^ analyzed using Mann–Whitney test; ^b^ analyzed using Independent T test; * statistically significant at *p* < 0.05.

## Data Availability

The raw data supporting the conclusions of this article will be made available by the authors on request.

## Abbreviations

The following abbreviations are used in this manuscript:

SBDP	Spectrin degradation products
EVD	Extra ventricular drainage
LD	Lumbar drainage
SAH	Subarachnoid hemorrhage
WFNS	World Federation of Neurological Surgery Scale
GOSE	Glasgow Outcome Scale Extended
